# Facial Expression Recognition Based on Weighted-Cluster Loss and Deep Transfer Learning Using a Highly Imbalanced Dataset

**DOI:** 10.3390/s20092639

**Published:** 2020-05-05

**Authors:** Quan T. Ngo, Seokhoon Yoon

**Affiliations:** Department of Electrical and Computer Engineering, University of Ulsan, Ulsan 44610, Korea; tanquan.dn@gmail.com

**Keywords:** facial expression recognition, deep convolutional neural network, transfer learning, auxiliary loss, weighted loss, class center

## Abstract

Facial expression recognition (FER) is a challenging problem in the fields of pattern recognition and computer vision. The recent success of convolutional neural networks (CNNs) in object detection and object segmentation tasks has shown promise in building an automatic deep CNN-based FER model. However, in real-world scenarios, performance degrades dramatically owing to the great diversity of factors unrelated to facial expressions, and due to a lack of training data and an intrinsic imbalance in the existing facial emotion datasets. To tackle these problems, this paper not only applies deep transfer learning techniques, but also proposes a novel loss function called weighted-cluster loss, which is used during the fine-tuning phase. Specifically, the weighted-cluster loss function simultaneously improves the intra-class compactness and the inter-class separability by learning a class center for each emotion class. It also takes the imbalance in a facial expression dataset into account by giving each emotion class a weight based on its proportion of the total number of images. In addition, a recent, successful deep CNN architecture, pre-trained in the task of face identification with the VGGFace2 database from the Visual Geometry Group at Oxford University, is employed and fine-tuned using the proposed loss function to recognize eight basic facial emotions from the AffectNet database of facial expression, valence, and arousal computing in the wild. Experiments on an AffectNet real-world facial dataset demonstrate that our method outperforms the baseline CNN models that use either weighted-softmax loss or center loss.

## 1. Introduction

Facial expressions are undoubtedly a dominant, natural, and effective channel used by people to convey their emotions and intentions during communication. Over the last few decades, automatic facial expression analysis has attracted significant attention, and has become one of the most challenging problems in computer vision and artificial intelligence fields. Numerous studies have been conducted on developing reliable automated facial expression recognition (FER) systems for use over a wide range of applications, such as human–computer interaction, social robotics, medical treatment, virtual reality, augmented reality, and games [1,2,3,4]. In this work, we constructed deep convolutional neural network (CNN)-based FER models to recognize eight common facial expressions (happy, sad, surprise, fear, contempt, anger, disgust, and neutral) from the AffectNet database of facial expression, valence, and arousal computing in the wild [5]. This was motivated by a upcoming challenge on AffectNet’s website [6].

The FER model is usually composed of three main stages: face detection, feature extraction, and emotion classification. First, the face and its components (e.g., eyes, mouth, nose, and eyebrows) are detected from images or video sequences. Then, features that are the most effective at distinguishing one expression from another are extracted from the face region. Finally, a classifier is constructed, given the extracted feature set for each target facial expression. The literature is rich with handcrafted face detection and feature extraction methods for FER that have achieved satisfactory results in laboratory-controlled settings [7,8,9,10,11]. However, these traditional methods have been reported to be incapable of discriminating a great diversity of unrelated factors in facial expressions (e.g., subtle facial appearances, head poses, illumination intensity, and occlusions) with FER tasks for in-the-wild settings [12,13].

Recently, the success of convolutional neural networks in both computer vision and pattern recognition has promoted a transition in FER from using handcrafted feature-learning methods to using deep learning technologies. A deep learning-based FER system commonly uses a CNN model to extract and learn high-level features directly from input images. Then, an output layer (which usually uses softmax as an activation function) is attached on top of the CNN model to distinguish the emotion to be detected. This allows a faster emotion recognition system with higher accuracy in challenging, real-world environments [14,15,16]. In this paper, we make use of the powerful feature-learning capacity of the deep CNN to build FER models. However, a few significant problems arise when applying deep learning to FER systems.

First, deep learning-based FER models require a large amount of data for training to acquire suitable values for model parameters. Directly training the FER model on small-scale datasets is prone to overfitting [17], which leads the model to be less generalized and incapable of handling FER tasks in real-world environments. Although a great effort has been made to collect facial expression training datasets, large-scale, annotated, facial expression databases are still limited [13]. Therefore, overfitting caused by a shortage of data remains a challenging issue for most FER systems.

Second, imbalances in the distribution of facial expression samples from real-world FER datasets may degrade the overall performance of the system [18]. Due to the nature of emotions, the number of collected facial images for the major classes (e.g., happiness, sadness, and anger) is much larger than for the minor classes (e.g., contempt, disgust, and fear). In the AffectNet dataset, happy category comprises about 47% of all the images, whereas contempt category comprises only 1.2%. FER systems being trained on an imbalanced dataset may perform well on dominant emotions, but they perform poorly on the under-represented ones. Usually, the weighted-softmax loss approach [5] is used to handle this problem by weighting the loss term for each emotion class based on its relative proportion in the training set. However, this weighted-loss approach is based on the softmax loss function, which is reported to simply force features of different classes to remain apart without paying attention to intra-class compactness. One effective strategy to address the problem of softmax loss is to use auxiliary loss to train the neural network. For instance, triplet loss [19] and center loss [20] introduce multi-loss learning to enhance the discriminating ability of CNN models. Although these loss functions do boost the discriminative ability of the conventional softmax loss, they usually come with limitations. Triplet loss requires a comprehensive process of choosing image pairs or triplet samples, which is impractical and extremely time-consuming owing to the huge number of pairs and samples in the training phase. Center loss does not consider inter-class similarity, which may lead to poor performance by the FER system. In addition, none of these auxiliary loss functions is able to address data-imbalance problems.

To address the first problem (the shortage of data), in this work, the transfer learning technique is applied to build the FER system. Transfer learning is a machine learning technique by which a model trained on one task is repurposed for another related task. It not only helps to handle the shortage of data but also speeds up training and improves the performance of the prediction model. In this paper, we take a transfer learning approach by employing two recent CNN architectures in two-stage, supervised pre-training and fine-tuning. Specifically, a squeeze-and-excitation network (SENet) model [21] which is pre-trained for the face identification task on the VGGFace2 [22] database from the Visual Geometry Group at Oxford University, was fine-tuned on the AffectNet dataset [5] to recognize the above-mentioned eight common facial expressions.

Tackling the second problem of imbalanced data distribution in existing FER datasets, we propose a new loss function called the weighted-cluster loss, which integrates the advantages of the weighted-softmax approach and the auxiliary loss approach. First, weighted-cluster loss learns a class center for each emotion, which simultaneously reduces the intra-class variations and increases the inter-class differences. Next, the proposed loss gives weights to each emotion class’s loss terms based on their relative proportion of the total number of samples in the training dataset. In other words, weighted-cluster loss penalizes networks more for misclassifying samples from minor classes while penalizing those networks less for misclassifying examples from major classes. Furthermore, the training process is simple because weighted-cluster loss does not require preselected sample pairs or triples.

Experiments were conducted to show the effectiveness of the proposed method. In addition to widely used metrics for classification (accuracy, F1-score [23], area under the receiver operating characteristic [ROC] curve [AUC] [24], and area under the precision-recall curve [AUC-PR] [25]), two measures of inter-annotator agreement (Cohen’s kappa [26] and Krippendorff’s alpha [27]) are used to evaluate the models. The experimental results with the AffectNet dataset [5] show that our transfer learning-based model with weighted-cluster loss outperforms other models that use either weighted softmax-loss or center loss.

In summary, the main contributions of this paper are listed as follows:

- First, the objective of this paper is to mitigate the overfitting problem caused by an insufficient amount of data and imbalanced data problem when building a FER model which recognizes eight common facial expressions on AffectNet dataset [5].
- Second, to address the overfitting problem, a deep transfer-based framework is proposed in which we utilize an SE-ResNet-50 model [21] (which was pre-trained on VGGFace2 data [22]) for fine-tuning on AffectNet dataset.
- Third, to alleviate the imbalanced-data problem, we propose a new loss function, named weighted-cluster loss, which gives weights to each emotion class’s loss terms based on their relative proportion of the total number of samples in the training dataset.
- Last, experiments are conducted to validate the effectiveness of the proposed method. The experimental results on AffectNet data show that our FER model outperforms its counterpart models in term of various evaluation metrics such as accuracy, F1-score, Cohen’s kappa score [26], Krippendorff’s alpha score [27], area under the receiver operating characteristic curve (AUC), and area under the precision-recall curve (AUC-PR).

The rest of this manuscript is organized as follows. Section 2 summarizes the existing literature related to facial expression recognition. Then, our proposed method is presented in detail in Section 3. The experiments with results discussion are presented in Section 4. Conclusions drawn from this work, in addition to possible future work are discussed in Section 5.

## 2. Related Works

This section summarizes recent studies in the literature that are related to facial expression recognition, deep transfer learning techniques used to solve FER tasks, and recent successful loss functions for training deep models.

### 2.1. Facial Expression Recognition Approaches

Over the last few decades, several approaches have been proposed to build FER models. Traditional methods mostly detect the face region and extract the geometry, appearance, texture, or other highlighted facial characteristics using handcrafted features and shallow learning, such as Gabor wavelet coefficients [7], Haar features [8], histograms of local binary patterns (LBPs) [9], LBPs on three orthogonal planes (LBP-TOP) [10], histograms of oriented gradients (HOG) [11], non-negative matrix factorization (NMF) [28], scale-invariant feature transform (SIFT) descriptors [29], and sparse learning [30]. Overall, these methods can solve the FER tasks in laboratory settings, where emotion images are produced in a controlled manner. However, with the introduction of relatively large real-world databases from emotion recognition competitions such as FER2013 [31] and emotion recognition in the wild [32,33,34,35,36], FER tasks have observed a big transition from laboratory-controlled setups to more challenging real-world scenarios. Traditional methods face many difficulties in solving inter-class similarity and intra-class differences, and are reported to be incapable of addressing the great diversity of factors unrelated to facial expressions.

Recently, the development of deep learning and the increase in the number of powerful CNN architectures [37,38,39] in the computer vision field implicitly promote using deep learning methods when building FER models. For example, the winner of the 2013 International Conference on Machine Learning [40] combined a deep CNN with a support vector machine (SVM) classifier to distinguish common emotions. However, despite the impressive feature learning ability of deep learning, difficulties remain when applied to FER such as the shortage of training data, the inter-class similarity, the intra-class differences, and the high imbalance of emotion classes appearing in most existing real-world facial expression databases. This work is taking advantage of deep models to extract robust facial features and translate them to recognize facial emotions. Table 1 summarizes the comparison between our approaches with existing studies. In this table, human resource refers to how much human labor needed to construct features learning/extraction model, computing resource refers to hardware resources needed to operate model, and computational complexity refers to operations per pixel.

### 2.2. Transfer Learning for Facial Expression Recognition

Studies in FER have suffered from the lack of data for training deep CNN models, which may have resulted in overfitting. To work around this problem, transfer learning has been widely used for facial recognition tasks. In fact, the use of auxiliary data can help FER models to obtain a high capacity without overfitting, thus improving the overall performance of the system. Usually, the weights of the CNN are initialized and pre-trained on additional data from other relative tasks (e.g., object detection and face recognition) before being fine-tuned using the target dataset. Clearly, applying transfer learning to the FER task has consistently achieved better results, compared to directly training the network on a small-scale FER dataset. Some popular datasets can be used as auxiliary data, such as ImageNet [41] for object recognition tasks and VGGFace from the Visual Geometry Group [42] for the face recognition task. In the work of Ly et al. [43], a Inception-ResNetV1 model was pre-trained on VGGFace2 [22] and AffectNet [5] then was used to develop a multi-modal 2D and 3D for real-word FER task. However, the number of existing 3D data for FER is limited thus make it difficult to construct a multi-modal FER model. Do et al. [44] used a ResNet-50 model pre-trained on VGGFace2 [22] as a feature extraction model. The model was then integrated with a LSTM [45] model to analyses facial expression on the video data.

It is worthwhile to note that in preliminary experiments, Ngo and Yoon showed the improvement in recognition performance when using ImageNet data [41] as auxiliary data for building a transfer learning-based FER model [46]. The authors fine-tuned a ResNet-50 [47] model, which was pre-trained with ImageNet data. However, the ImageNet dataset was developed for object detection task, which may not be sufficiently related to the FER task. In this paper, to build the transfer learning-based FER model, we employ the more advanced CNN architecture (i.e., SENet [21]) and then fine-tune it for FER task. Note that this transferred model is pre-trained with VGGFace2 data [22] for the face identification task which is more related to the FER task than object detection tasks. This may help improve the performance of the transfer learning-based FER system. Furthermore, in [46], authors simply fine-tuned their models using softmax loss and did not consider the imbalanced data problem, which degraded the recognition performance of FER system. In this paper, we focus on handling the shortage of data as well as tackling the imbalanced data problem by applying weighted loss and auxiliary loss approaches.

### 2.3. Data Re-Sampling and Augmentation

As the nature of emotions, facial expression data collected in real-world settings are highly imbalanced in the number of samples in each class (e.g., the number of images in the happy class is much greater than the number of images that show disgust). Using imbalanced data for training may degrade the performance of FER models [18]. Data re-sampling and generating samples using generative adversarial networks (GAN) [48] are usually considered as solutions to mitigate the imbalanced data problem. Mollahoseini et al. [5] used down-sampling and up-sampling methods to balance the distribution of data in the training set, which alleviates the imbalanced data problem. However, the two data re-sampling methods simply randomly reduce the number of samples in major classes or duplicate samples in minor classes without actually collecting further data. Under-sampling may discards samples that could be important for the model while over-sampling significantly increases the model training time [49]. Lai et al. [50] proposed a GAN that generates frontal face images from input non-frontal face images. This model can be employed to augment more data for training FER models. Nonetheless, the reliability of the new data needs to be carefully verified before being used to train FER models, otherwise, the data may degrade the performance of FER systems. Our solution is based on a weighted loss approach that tackles the imbalanced data problem without the need of data re-sampling or augmentation step.

### 2.4. Weighted Loss and Auxiliary Loss

Weighted-softmax loss gives weights to the loss terms of each emotion class based on its relative numbers of samples in the training set. In this way, the loss function heavily penalizes the FER model for misclassifying examples from minor classes, while lightly penalizing the model for misclassifying examples from major classes. Mollahoseini et al. [5] showed that the weighted-loss methods achieve the highest performance and outperform the re-sampling methods. However, since the weighted-softmax loss function is built based on conventional softmax loss, it inherits the limitations of softmax loss. For example, weighted-softmax loss simply forces features of different classes to remain apart, without paying attention to intra-class compactness.

To tackle the limitations of softmax loss, several auxiliary loss approaches have been proposed, and they can be used to improve the discriminative ability of FER models. Contrastive loss [51] inputs the CNN model with a pair of training samples, which forces the features of same-class pairs to be as similar as possible while requiring a pairwise distance longer than a predefined margin if the input samples belong to different classes. Similar to contrastive loss, triplet loss [19] encourages a distance constraint between samples that come from different classes. Specifically, triplet loss requires one positive sample to be closer to a preselected anchor than one negative sample with a fixed margin. Based on triplet loss, there are two variations proposed to support softmax loss. (N + M)-tuples cluster loss [52] mitigates the difficulty of anchor selection and threshold validation, whereas exponential triplet-based loss [53] gives more weight to difficult samples during an update of network parameters. In spite of some success, these loss functions still need a careful pre-selection process. However, this becomes impossible when the number of training samples is relatively large. Recently, center loss [20] has been proposed for the face recognition task; it penalizes the distance between deep features and their corresponding class clusters in order to reduce intra-class differences. However, center loss does not take inter-class similarity into account. Motivated by this, island loss [54] was proposed to simultaneously force deep features of a sample close to its cluster, and to push class cluster centers far away from each other to enlarge the distance between samples of different expressions. Nonetheless, adding more loss terms usually comes with difficulty in the training phase caused by extra hyper-parameters that need to be adjusted. In addition, none of these aforementioned loss functions take into consideration the imbalance in the number of training samples from each emotion class in the training dataset. This may lead FER models to perform poorly with minority classes, even though they perform well with majority classes.

By analyzing the complementary nature of weighted loss and center loss, we propose a new loss function, named weighted-cluster loss, which not only takes highly skewed facial emotion data into consideration, but it also uses multiple loss terms to improve the performance of the FER models.

## 3. Methods

**Figure 1:**
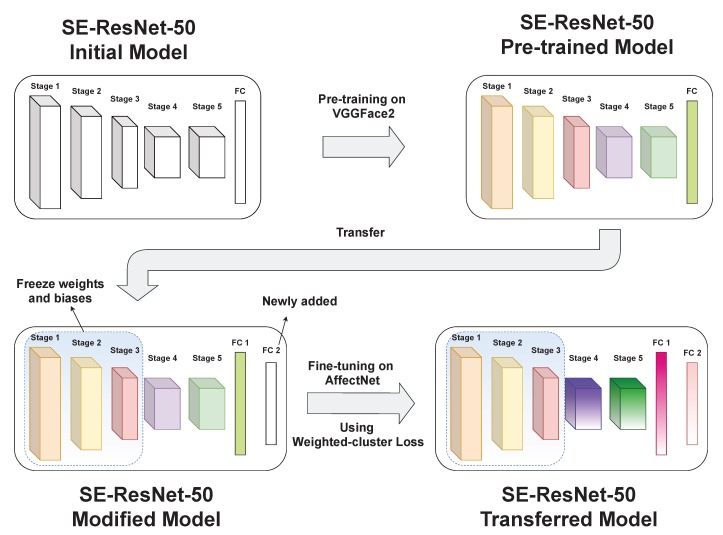
Framework of the proposed method. A SE-ResNet-50 model [21], which was pre-trained on VGGFace2 data [22] for face identification, is fine-tuned with AffectNet data [5] for facial recognition using weighted-cluster loss. Before the fine-tuning phase, we add one more fully connected layer to the model while froze the three first stages of the pre-trained model to save computing power. The weighted-cluster loss is used at the output layer to update model parameters. Best view in color.

In this section, we first describe our deep transfer–learning framework and then propose weighted-cluster loss. The framework of the proposed method is shown in Figure 1.

### 3.1. Base Model and Pre-Training

#### 3.1.1. Base Models

Convolutional neural networks have achieved great success in the fields of pattern recognition and computer vision. This motivated us to base our FER models on a recent representative CNN architecture in the computer vision field. In this work, we employed the SE-ResNet-50 model [21], which is the ResNet-50 model [47] integrated with SE-ResNet modules as our base model. This CNN architecture uses SE modules that integrated with ResNet CNN architectures and improved the feature learning capacity of the integrated model. A wide range of experiments showed the effectiveness of SENets that achieve state-of-the-art performance across multiple datasets and tasks. This was demonstrated for object and scene classification, with a squeeze-and-excitation network winning the ILSVRC 2017 competition.

#### 3.1.2. Pre-Training

This work uses publicly available pre-trained model of the base model (i.e., transfer learning-based model) instead of directly pre-training it. Specifically, the SE-ResNet-50 pre-trained model, which is published on the official website of the VGGFace2 database [55], was trained with the VGGFace2 dataset [22] for the task of recognizing 8631 faces. As a results, the network learned rich feature represent for face factors.

### 3.2. Fine-Tuning

In this stage, we add one more fully connected layer to the pre-trained SE-ResNet-50 model (before the output layer; see Table 2) and we then changed the output layer to eight-class classification. In addition, we froze the weights and biases of Stage 1 to Stage 3 on the pre-trained model. This process is usually found in many transfer learning-based systems when computing power is limited. Last we fine-tuned the adjusted model with the AffectNet dataset [5] to recognize the eight facial expressions (happy, sad, surprise, fear, contempt, anger, disgust, and neutral). Specifically,

### 3.3. Weighted-Cluster Loss

#### 3.3.1. Review of Weighted-Softmax Loss

Weighted-softmax [5] often comes as a solution for tackling the imbalanced dataset problem. Weighted-softmax loss weighs the entropy loss of each of the emotion classes by their relative proportion of the total number of samples in the training dataset. The entropy weighted-softmax loss for the $i^{th}$ training sample is defined as:

(1) $$L_{\mathrm{Weighted}\text{-}\mathrm{softmax}}=-\sum_{i=1}^{m}W_{y_{i}}\log\left(\hat{p_{i}}\right)$$

where *m* is the number of training samples in the mini batch (i.e., the batch size), $y_{i}$ is the class label of the $i^{th}$ training sample, $W_{y_{i}}$ denotes the weight assigned to the class where the label $y_{i}$, and $\hat{p_{i}}$ is the softmax-predicted probability of the $i^{th}$ sample.

Although weighted-softmax loss is able to tackle the imbalanced dataset problem, it still has some limitations. Since weighted loss simply adds weight to the conventional softmax loss, it is incapable of handling not only high inter-class similarity but also high intra-class variations.

#### 3.3.2. Review of Center Loss

Center loss [20] can be incorporated with conventional softmax loss to reduces the intra-class variations by compressing samples towards their corresponding class center in the feature space during training. Center loss for the $i^{th}$ training sample is defined as:

(2) $$L_{\mathrm{Center}}=\frac{1}{2}\sum_{i=1}^{m}{∥x_{i}-c_{y_{i}}∥}_{2}^{2},$$

where $L_{\mathrm{Center}}$ denotes the center loss, $x_{i}\in R^{d}$ denotes the deep feature of the $i^{th}$ training sample (i.e., taken from the last fully connected layer, before the output layer), and $c_{y_{i}}\in R^{d}$ denotes the center of class label $y_{i}$; d is the feature dimension.

To train the CNN model, joint supervision of softmax loss and center loss is employed as follows:

(3) $$L=L_{\mathrm{Softmax}}+\lambda L_{\mathrm{Center}}$$

where *L* denotes the total loss, $L_{\mathrm{Softmax}}=-\sum_{i=1}^{m}\log\left(\hat{p_{i}}\right)$ is the the entropy softmax loss and λ is a scalar used for balancing the two losses.

Since inter-class similarities are ignored by the center loss, the class clusters may move closer to, or even overlap, each other. Furthermore, center loss was not proposed to deal with the imbalanced dataset problem. Due to dataset imbalances, the centers of major emotion classes are more frequently updated than those of minor classes, which leads to poor performance of FER model on the minor classes.

#### 3.3.3. The Proposed Weighted-Cluster Loss

To address the limitations of existing loss functions (e.g., weighted-softmax loss and center loss), we propose a new loss function called weighted-cluster loss. It effectively tackles the imbalanced data problem by taking into consideration the imbalanced proportion in the number of samples of each emotion class in the training set. Furthermore, weighted-cluster loss adds a new term to center loss, which simultaneously pulls the centers of each class apart. This may allow the model to simultaneously handle the high intra-variation and the inter-class similarity in the FER dataset. The weighted-cluster loss for the $i^{th}$ training sample is given by Equation (4):

(4) $$L_{\mathrm{Weighted}\text{-}\mathrm{cluster}}=\frac{1}{2}\sum_{i=1}^{m}W_{y_{i}}\frac{{∥x_{i}-c_{y_{i}}∥}_{2}^{2}}{\left(\sum_{j=1,j\neq i}^{k}{∥c_{j}-c_{y_{i}}∥}_{2}^{2}\right)+\alpha }$$

where $W_{y_{i}}$ denotes the weight assigned for the class where the label is $y_{i}$, *k* denotes the number of emotion classes, and the constant, α, prevents the denominator from equaling 0. In this paper, we set α=1 by default.

In Equation (4), the numerator penalizes the distance between the deep feature of the training sample (i.e., taken from the last fully connected layer before the output layer) and its corresponding center, and the denominator penalizes the distance between the corresponding center and all other class centers. By minimizing weighted-cluster loss, the deep features of training samples from the same class (i.e., the cluster) will be compacted in the feature space, whereas the distance between different classes of clusters will be enlarged. In addition, weight $W_{y_{i}}$ is used to tackle the imbalanced dataset problem by penalizing the models less for misclassifying samples from majority classes while heavily penalizing the model for misclassifying samples from minority classes.

The overall loss function of FER training is given by:

(5) $$\begin{array}{cc} L & =L_{\mathrm{Weighted}\text{-}\mathrm{softmax}}+\lambda L_{\mathrm{Weighted}\text{-}\mathrm{cluster}}\\ \; & =-\sum_{i=1}^{m}W_{y_{i}}\left(\log(\hat{p_{i}})-\frac{\lambda }{2}\frac{{∥x_{i}-c_{y_{i}}∥}_{2}^{2}}{\sum_{j=1,j\neq i}^{k}{∥c_{j}-c_{y_{i}}∥}_{2}^{2}+\alpha }\right) \end{array}$$

where λ is used to balance the two losses.

In this work, we define $W_{y_{i}}$ as:

(6) $$W_{y_{i}}=\frac{N_{\min}}{N_{y_{i}}}$$

where $N_{\min}$ denotes the number of samples from the smallest class (i.e., disgust), and $N_{y_{i}}$ denotes the number of samples from the class where the label is $y_{i}$.

The joint weighted-cluster loss and weighted-softmax loss can be directly used for training deep neural networks. The class centers, $c_{y_{i}}$, are updated each iteration through the training process. Specifically, the class centers are updated based on mini-batches, and use a scalar γ to control the learning rate of class centers (and in our experiments, we set γ=1). The partial derivative of the weighted cluster loss, $L_{\mathrm{Weighted}\text{-}\mathrm{cluster}}$, with respect to the sample’s feature, $x_{i}$, can be calculated as follows:

(7) $$\frac{\partial L_{\mathrm{Weighted}\text{-}\mathrm{cluster}}}{\partial x_{i}}=W_{y_{i}}\frac{x_{i}-c_{y_{i}}}{\left(\sum_{l=1,l\neq i}^{k}{∥c_{l}-c_{y_{i}}∥}_{2}^{2}\right)+\alpha }$$

The update of the $j^{th}$ class center can be calculated with Equation (8):

(8) $$\Delta c_{j}^{t}=\sum_{i=1}^{m}W_{y_{i}}\frac{\delta \left(y_{i}=j\right)\left(x_{i}-c_{j}\right)}{\left(\sum_{l=1,l\neq i}^{k}{∥c_{l}-c_{y_{i}}∥}_{2}^{2}\right)+\alpha }$$

where $\delta (y_{i}=j)=1$ if $y_{i}=j$, and $\delta (y_{i}=j)=0$ if $y_{i}\neq j$.

Then, the class centers can be updated in each mini-batch with a learning rate γ:

(9) $$c_{j}^{t+1}=c_{j}^{t}-\gamma \Delta c_{j}^{t}$$

In Algorithm 1, we summarize the learning process of the FER model with the joint loss functions.
**Algorithm 1** Learning algorithm of the FER model with the jointly loss functions**Input:** Training data $\left\{x_{i}\right\}$, mini-batch size *m*, number of iterations *T*, learning rates μ and γ, and hyper-parameters λ1:**Initialize:** Network layer parameters *W*, weighted-softmax loss parameters θ, and weighted-cluster loss parameters (i.e., centers) $\left\{c_{j}|j=1,2,\ldots ,n\right\}$.2:**for**t=1 to *T* **do**3:     Calculate the joint loss4:          $L^{t}=L_{\mathrm{Weighted}\text{-}\mathrm{softmax}}^{t}+\lambda L_{\mathrm{Weighted}\text{-}\mathrm{cluster}}^{t}$5:     Compute the backpropagation error for each *i*6:          $\frac{\partial L^{t}}{\partial x_{i}^{t}}=\frac{\partial L_{\mathrm{Weighed}\text{-}\mathrm{softmax}}^{t}}{\partial x_{i}^{t}}+\lambda \frac{\partial L_{\mathrm{Weighted}\text{-}\mathrm{cluster}}^{t}}{\partial x_{i}^{t}}$7:     Update the weighted-softmax loss parameters θ8:          $\theta^{t+1}=\theta^{t}-\mu \frac{\partial L_{\mathrm{Weighed}\text{-}\mathrm{softmax}}^{t}}{\partial \theta_{i}^{t}}$9:     Update the weighted-cluster loss parameters $c_{j}$ for each *j* as Equation (9)10:          $c_{j}^{t+1}=c_{j}^{t}-\gamma \Delta c_{j}^{t}$11:     Update the network parameters *W*12:          $W^{t+1}=W^{t}-\mu \frac{\partial L^{t}}{\partial x_{i}^{t}}\frac{\partial x_{i}^{t}}{\partial W^{t}}$13:**end for****Output:** Network layer parameters *W*

## 4. Experiments

### 4.1. Experimental Datasets

#### 4.1.1. AffectNet Dataset

**Figure 2:**
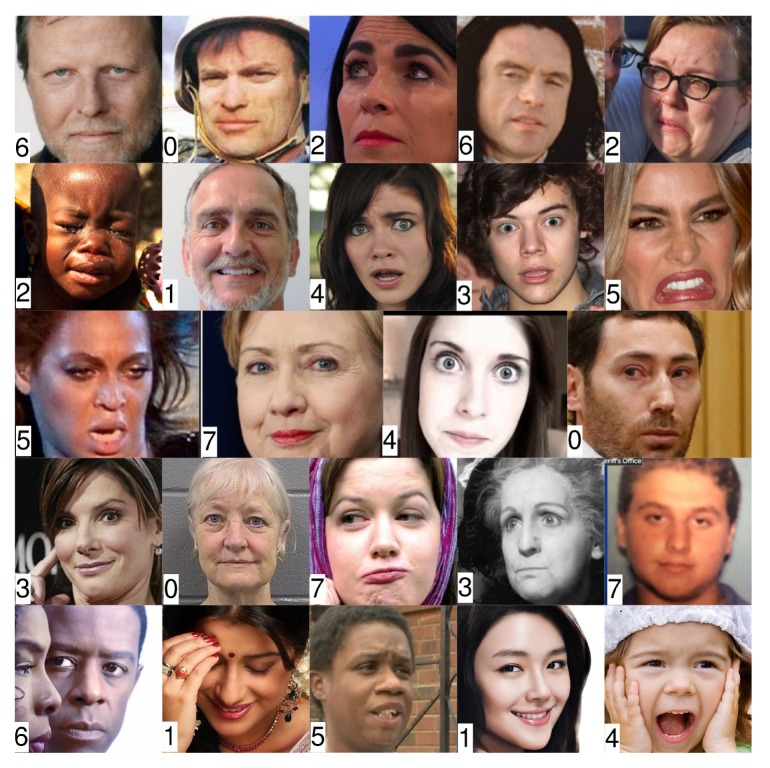
Sample images from the AffectNet dataset (0: neutral; 1: happy; 2: sad; 3: surprise; 4: fear; 5: disgust; 6: anger; 7: contempt).

In this work, we consider the problem in recognizing eight common facial expressions from the AffectNet dataset [5]. The AffectNet dataset contains more than 1,000,000 images from the Internet that were obtained by querying different search engines using emotion-related tags. AffectNet is by far the largest database that provides facial expressions in two different emotion models (a categorical model and a dimensional model), which can be used for studies in automated recognition of facial expressions, valences, and arousal in real-world scenarios. About 450,000 images already have manually annotated labels for eight basic expressions which are neutral, happy, sad, surprise, fear, disgust, anger, and contempt, as well as some non-emotion–related image classes such as none, uncertain, and non-face. Figure 2 shows some sample images from the dataset.

In this work, only images of the eight common emotion classes (manually annotated) were used to train the FER model. From each of the eight emotion classes, we randomly selected 500 samples for a validation set and another 500 samples were selected for a test set. The remainder were used for fine-tuning the FER models. The numbers of samples in the training, validation, and test sets are shown in Table 3.

**Figure 3:**
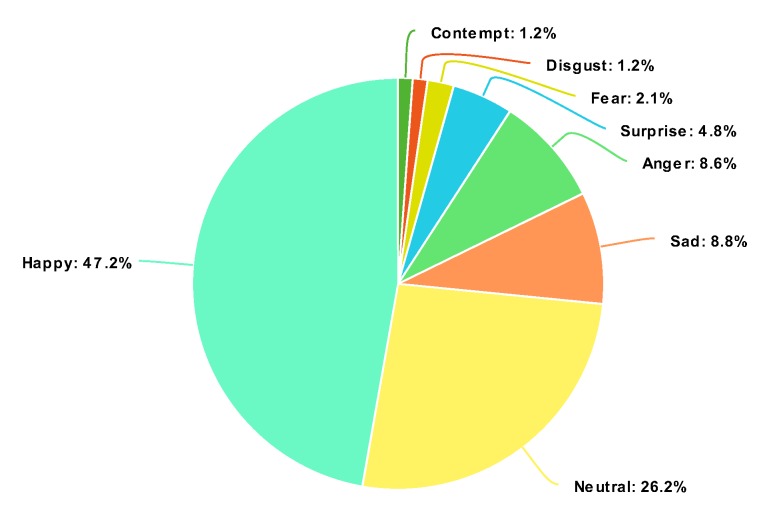
Distribution of the eight classes in the training set.

Although AffectNet is considered one of the largest facial expression databases, it still has shortcomings when used for training FER models. This database is highly imbalanced, as can be seen in Figure 3. Specifically, as seen in Table 3, the number of images in the largest category (happy with 134,915 images) is approximately 30 times larger than the smallest category (contempt, with 4250 images). Furthermore, images were manually annotated, which may result in a low-reliability dataset. Therefore, transfer learning is still needed to mitigate these drawbacks.

#### 4.1.2. VGGFace2 Dataset

**Figure 4:**
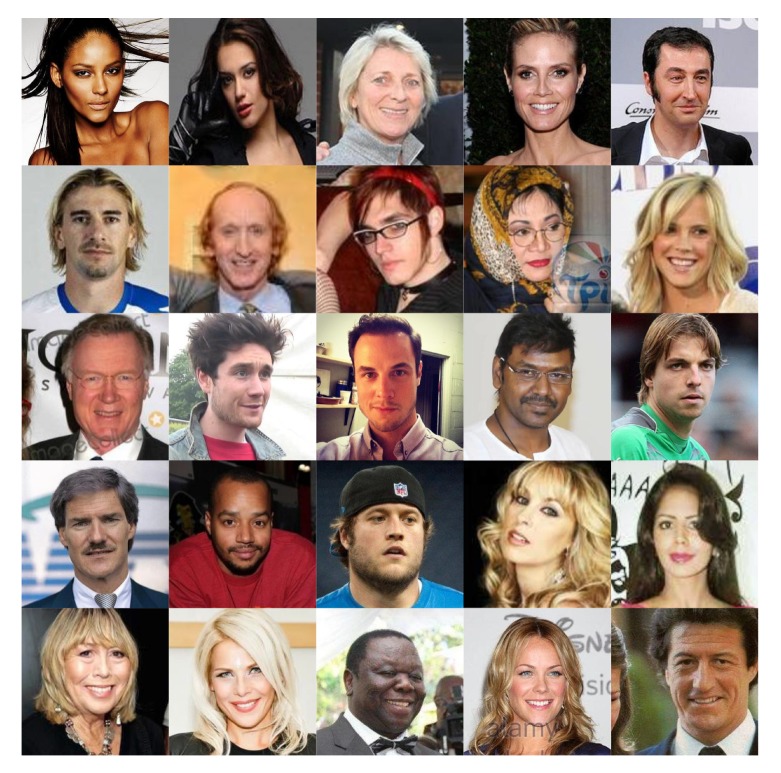
Sample images from the VGGFace2 dataset.

VGGFace2 [22] is a new large-scale face dataset that contains 3.31 million images of 9131 subjects, with an average of 362 images for each subject. Images were downloaded from Google’s image search function, and they have large variations in pose, age, illumination, ethnicity, and profession (e.g., actors, athletes, politicians). Figure 4 shows some sample images from the VGGFace2 dataset.

### 4.2. Evaluation Metrics

There are various evaluation metrics in the literature to measure discriminative performance of the FER model. In addition to several widely used metrics for classification, such as accuracy, F1-score [23], area under the ROC curve (AUC) [24], and area under the precision–recall curve (AUC-PR) [25], two measures of inter-annotator agreement (Cohen’s kappa [26] and Krippendorff’s alpha [27]) are used in our work. In statistics, Cohen’s kappa measures inter-rater reliability, which is the degree of agreement among raters, given the same data. Krippendorff’s alpha (also called Krippendorff’s coefficient) is an alternative to Cohen’s kappa for determining inter-rater reliability. Table 4 lists acronyms used in this paper.

### 4.3. Experiment Setups and Implementation Details

We experimented with different schemes to point out the effectiveness of using transfer learning as well as the proposed loss function for FER tasks. In detail, we fine-tune the transfer learning-based model (e.g., SE-ResNet-50 pre-trained with VGGFace2 dataset) with different loss settings, such as the conventional softmax loss, center loss with softmax loss [20], weighted-softmax loss [5], center loss with weighted-softmax loss (i.e., replace softmax loss by weighted-softmax loss), and the proposed weighted-cluster loss with weighted softmax loss. In addition, to evaluate the effectiveness of transfer learning, we also trained the base model using only the AffectNet training dataset with the same loss settings as those used for transfer learning-based model. In all experiments, we set the λ value (i.e., the scalar used to balance the loss terms) to 0.5 when using center loss, and to 1.0 when using weighted-cluster loss.

The pre-trained model was fine-tuned using the stochastic gradient descent algorithm with hyper-parameters (momentum = 0.9, weight decay = 0.0005). Note that we fine-tuned the pre-trained CNN model using a much smaller dataset (AffectNet compared with VGGFace2), and thus, the initial learning rate was set to 0.001, which is lower than the typical value of 0.01, in order to not drastically alter the pre-trained weights. The learning rate was dropped by a factor of 2 following every 10 epochs of training. For the base models that were trained using only AffectNet data (i.e., no pre-training), the initial learning rate was set to 0.01, and all other settings were kept the same. All experiments were implemented using the PyTorch library and were trained on a four-core Xeon CPU with a single Titan-XP GPU. Batch size for fine-tuning the transfer learning-based models was set to 36, while the batch size for training the base models from scratch was set to 30.

To enrich the scale of the dataset and mitigate the overfitting problem, it is necessary to conduct data augmentation. During the fine-tuning phase, input images were randomly cropped and resized to 224 × 224 pixels; the horizontal flip was randomly extracted from the cropped images. In addition, before being input into the FER model, all input samples were normalized by using the ImageNet mean and standard deviation (std) (mean = [0.485, 0.456, 0.406], std = [0.229, 0.224, 0.225]), which is a common practice for deep CNN models when working with RGB images.

### 4.4. Results and Discussions

Table 5 shows the accuracy, F1-score, Cohen’s kappa, Krippendorff’s alpha, AUC, and AUC-PR of different FER models on the test set. These values are averages over the eight classes. All metrics except for accuracy were calculated in a binary-class manner, whereas accuracy is defined in a multi-class manner. From the results in Table 5, we have the following observations.

First, the proposed FER model achieved the highest performance in terms of all evaluation metrics, outperforming their counterparts. In detail, model 10 (the transferred SE-ResNet-50 model fine-tuned using the proposed joint weighted-cluster and weighted-softmax) achieved recognition accuracy of 60.70%. They led the second-best model (i.e., model 8) by approximately 1% in terms of recognition accuracy. As reported in [5], the average agreement over eight emotion categories between two human annotators (randomly chosen out of a total of 12 annotators) on only a part of the AffectNet data was only 65.56%, which may be considered the maximum achievable recognition accuracy. This emphasizes the fact that recognizing human emotions from facial expressions in a real-world scenario is a challenging task, and the newly proposed weighted-cluster loss function is capable of addressing challenging factors such as subtle facial appearance, head pose, illumination intensity, and occlusions.

In terms of F1-score, the proposed model (model 10) surpassed its counterparts. In most imbalanced classification problems (e.g., facial expression recognition tasks on an imbalanced dataset like AffectNet), F1-score, which is the weighted average of precision and recall, gives a better measure of incorrectly classified cases than the accuracy metric. In addition, model 10 also achieved the best kappa, alpha, and AUC-PR values, which outperforms models that use other loss functions. Similar to F1-score, these values give us alternatives to accuracy when measuring the reliability of automated FER systems. This shows that the proposed FER model is dependable when it comes to solving facial expression recognition problems in a real-world scenario. It should be noted that model 8 achieves the best AUC value. This is because model 8 achieves good performance on the positive class (high AUC) at the cost of a high false negatives rate. On the other hand, the proposed model (model 10) tries to reduce the false negatives rate while maintaining good performance on the positive class.

Second, under the supervision of the same loss functions, transferred FER models performed better than FER models that are trained from scratch (i.e., models 1, 2, 3, 4, and 5, vs. models 6, 7, 8, 9, and 10, respectively). This shows the benefit of using transfer learning where the pre-trained model, which has the ability to learn features for face identification, can be transferred to recognize facial expressions to improve the recognition performance. The proposed model (model 10) which integrated both transfer learning and weighted loss approaches thus achieved the highest performance.

Next, the performance of model 1 and model 6 (models using conventional softmax loss) are lower than the performance model 5 and model 10 (models using weighted-cluster loss) by large margin (50.65% and 52.22% vs. 56.27% vs. 60.70%). This is because the softmax loss is incapable to handle the imbalanced data problem which leads to the poor performance of FER model on minor classes (e.g., contempt, disgust). In contrast, the proposed weighted-cluster alleviates the imbalanced data problem by giving weight to the loss term of each class. This help improve performance of model on small classes. In addition, center loss-based models (models that were fine-tuned using joint center loss with either softmax loss or weighted-softmax loss) performed worse than the models fine-tuned using only softmax loss or weighted-softmax loss. The recognition accuracy of model 7 which uses center with softmax loss function is only 46.07%, which is even lower than that of model 6 (using softmax loss). Similar phenomena can be found in the case of model 9. Model 9 that using center loss with weighted-softmax loss achieves lower accuracy than model 8 that using only weighted-softmax. This shows that the existing center loss is not suitable for tackling data imbalance problems where the centers of the major emotion classes are updated more frequently than the centers of minor emotion classes.

Last, model 10 (transferred model that was fine-tuned using the proposed joint weighted-cluster loss and weighted-softmax loss) achieved better performance compared to model 9 (transferred model that was fine-tuned using joint center loss and weighted-softmax loss) in terms of all evaluation metrics (e.g., in terms of accuracy: 60.70% vs. 59.60%, respectively). This shows that the proposed cluster loss effectively handles the limitations of center loss and weighted-softmax loss by not only taking the imbalanced data into consideration but by also simultaneously improving intra-class compactness and enlarging inter-class differences.

**Figure 5:**
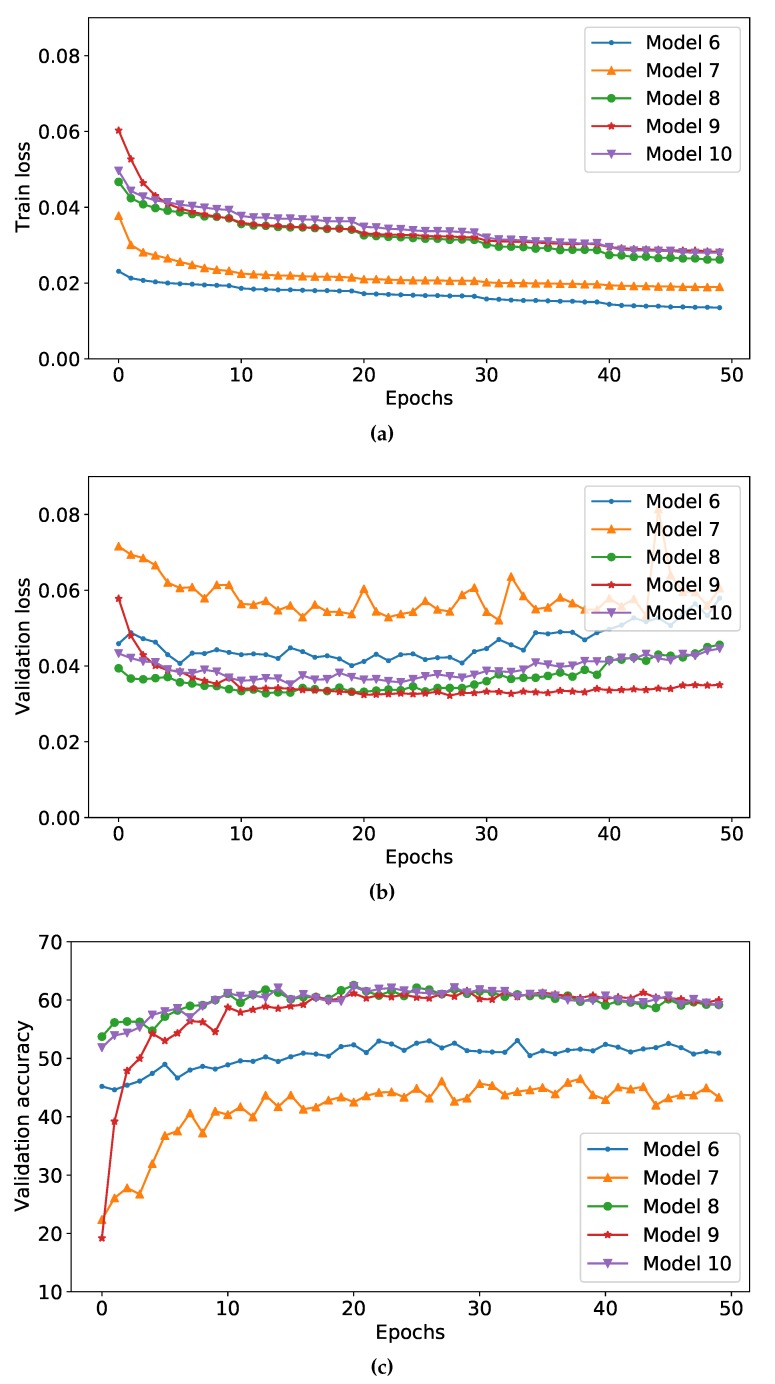
Learning curves of different models over number of epoch trained. (**a**) Train loss during the training. (**b**) Validation loss during the training. (**c**) Validation accuracy during the training. Best view in color.

Figure 5 shows the training loss and the validation accuracy of different models during the training phase. We can see that the training loss of auxiliary loss-based models (i.e., models 8, 9, and 10) is higher than the model using only softmax loss or weighted-softmax loss. This is because we added more loss terms to the total loss function. During the training phase, we can observe that all models are convergent as epochs are trained.

To compare our model with existing models, we also conducted experiments in [46] where authors used a ResNet-50 model [47] which is pre-trained with ImageNet data [41] for object detection task as their base model and then fine-tuned it with AffectNet data for FER task. It is worthwhile to note that, in [46], authors fine-tuned their model using only the conventional softmax loss. In our experiments, we further fine-tuned the ResNet-50 model (pre-trained with ImageNet) using the same loss settings that were used to fine-tune SE-ResNet-50 model (e.g., weighted-softmax loss, center with softmax loss, and so forth). Recognition performance of ResNet-50-based models on are shown in Table 6.

As can be seen in Table 6, the transferred ResNet-50 model that was fine-tuned using the proposed weighted-cluster loss (i.e., model 20) outperforms its counterpart models that were fine-tuned using other loss functions. This strengthens the point that the proposed weighted-cluster loss function is capable to handle the imbalanced data problem in the AffectNet dataset.

By comparing the performance of our models (Table 5) with the performance of the existing models in [46] (Table 6), we have following observation. Using the same loss function, our models (i.e., model 6, 7, 8, 9, 10) that used the SE-ResNet-50 model achieve better recognition performance than the models proposed in [46] (i.e., model 16, 17, 18, 19, 20, respectively) that used the ResNet-50 model pre-trained for object detection. For example, in terms of recognition accuracy, our best fine-tuned model (i.e., model 10) leads the best fine-tuned model that used the ResNet-50 model (i.e., model 20) about 1.25% (e.g., 60.70% vs 59.45%). This is because we used a more advanced CNN architecture (i.e., SE-ResNet-50) that was then pre-trained for face identification, instead of object detection.

**Figure 6:**
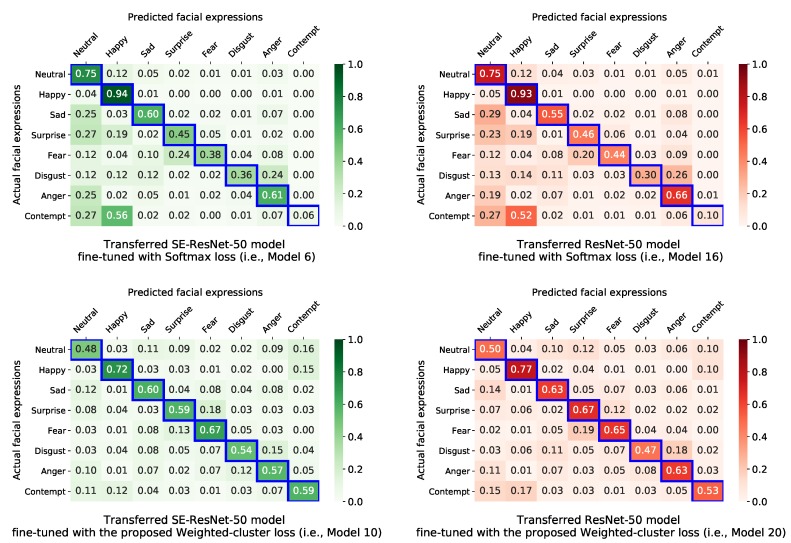
Confusion matrices of the transfer learning-based models on the test set.

To investigate the effectiveness of the proposed weighted-cluster loss function when handling the imbalanced dataset problem, we further plotted in Figure 6 the confusion matrices of the transfer learning-based models that were fine-tuned using the conventional softmax loss (model 1 and model 6) and the proposed joint weighted-cluster loss and weighted-softmax loss (model 10 and model 20).

We can see that weighted-cluster loss effectively solved the high imbalanced data problem with the AffectNet dataset. In particular, the FER performance with minor emotion classes dramatically improved. For example, recognition accuracy of the transferred SE-ResNet-50 model for the contempt class improved by approximately 1000%—from 6% when fine-tuned using the softmax loss, to 59% when fine-tuned using the proposed weighted-cluster loss. For the disgust class, it improved by about 150%—from 36% when fine-tuned using the softmax loss to 54% when fine-tuned using the proposed weighted-cluster loss. This is because the proposed loss function penalizes misclassifying samples from these classes more. However, the FER performance with major emotion classes (e.g., happy and neutral) slightly decreased, because the loss function may not sufficiently penalize misclassifying samples from these classes. The same trend can be found in case of the transferred ResNet-50 model where the recognition accuracy of class contempt increases about 500% (from 10% to 53%) and that of class disgust increases about 150% (from 30% to 47%).

It is worthwhile to note that although the weighted-cluster loss tries to increase the inter-class difference, the similarities between some emotion classes such as happy vs. contempt, neutral vs. contempt, surprise vs. fear, and disgust vs. anger still remain to some extent. This is due to the natural similarity between these emotion classes. For this reason, there were up to 16% samples of the neutral class and and 15% samples of happy class that was misclassified as the contempt class by model 10. In the opposite direction, there was also a high percentage (e.g., 11% and 12%) samples of the contempt class misclassified as the neutral and happy classes, respectively. Similarly, in model 10, 18% samples of surprise class was misclassified as the fear class, and 13% samples of the fear class was misclassified as the surprise class while the disgust and anger classes were falsely recognized as the other at the rate of 15% and 12%, respectively.

### 4.5. Threats to Validity

- Threat to internal validity: Threat to internal validity include errors when implementing the codes and conducting experiments. Although the implementations and experiments were carefully verified, errors are possible.
- Threat to external validity: Threats to external validity include the generalization to other datasets of the results in this paper. Applying the proposed methods in this paper to additional datasets will reduce this threat.
- Threats to construct validity: Threats to construct validity include the appropriateness of the benchmark algorithms and evaluation metrics. For the most part, the proposed FER model outperformed their counterparts on the same test dataset as expected. The evaluation metrics used for validating models (i.e., accuracy, F1-Score, Kappa score, AUC, and AUC-PR) are common in machine learning studies to evaluate the performance of classification models.

## 5. Conclusions

This paper proposed a facial expression recognition model based on deep transferred learning and a novel weighted-cluster loss to mitigate the shortage an imbalanced data problems. An SE-ResNet-50 model which is pre-trained for face identification task is fine-tuned to recognize eight common facial expressions in AffectNet data. This not only helps to save computing resource but also alleviate the shortage of training data problem. Then, the proposed weighted-cluster loss was used in the fine-tuning phase to tackle the high imbalance in data distribution of AffectNet data. Multiple metrics have been used to evaluate the effectiveness of the proposed model. Experimental results on the test set indicate that the proposed FER model can outperform its counterpart models which uses either weighted-softmax loss or center loss. However, the proposed model is built to recognize facial expressions on static image data, which may limit its applicability. Moreover, using generative adversarial networks (GAN) to generate more data for training FER models is considered as our other future work.

## Figures and Tables

**Table 1:** Comparison between FER approaches.

Approach	Method	Human Resource	Computing Resource	Computational Complexity	Accuracy
Conventional	Gabor wavelets coefficients [7]	High	Low	Low	Medium
	Haar features [8]	High	Low	Low	Medium
	Local binary pattern (LBP) [9]	High	Low	Low	Medium
	LBP on three orthogonal planes (LBP-TOP) [10]	High	Low	Low	Medium
	Scale-invariant feature transform (SIFT) [29]	High	Low	Low	Medium
	Histogram of gradient (HOG) [11]	High	Low	Low	Medium
Deep learning-based	Convolutional neural network [40]	Low	High	High	High
	Transfer learning-based CNN (Our approaches)	Low	Medium	Medium	High

**Table 2:** Detailed architectures of SE-ResNet-50 in the fine-tuning phase. Convolution blocks (SE-ResNet-50 uses the SE-ResNet block [21]) are shown in brackets, with the numbers of blocks stacked.

	Output Size	Kernel	Repeat
Stage 1 (Freeze)	112×112	convolution 7×7	1
Stage 2 (Freeze)	56×56	convolution block	3
Stage 3 (Freeze)	28×28	convolution block	4
Stage 4	14×14	convolution block	6
Stage 5	7×7	convolution block	3
	1×1	global average pooling	
Fully connected	[2048×512] Fully connected		
Fully connected	[512×8] Fully connected		
Output layer	8-d softmax		

**Table 3:** Numbers of samples in training, validation, and test sets.

Emotion	Training	Validation	Test
Neutral	74,374	500	500
Happy	133,915	500	500
Sad	24,959	500	500
Surprise	13,590	500	500
Fear	5878	500	500
Disgust	3303	500	500
Anger	24,382	500	500
Contempt	3250	500	500
Total	283,651	4000	4000

**Table 4:** List of acronyms.

Abbreviation	Meaning
ResNet	Residual neural network
SE-ResNet	ResNet-based squeeze and excitation neural network
Alpha	Krippendorff’s alpha score
Kappa	Cohen’s kappa score
AUC	Area under the receiver operating characteristic curve
AUC-PR	Area under precision recall curve

**Table 5:** Recognition performance of different FER models on the test set.

Base Model	Model No.	Pre-Trained	Loss Function	Accuracy	F1-Score	Kappa	Alpha	AUCPR	AUC
SE-ResNet-50	1	No	Softmax	50.65	46.87	43.60	42.60	62.87	90.67
	2	No	Center with softmax	46.07	39.24	38.37	36.89	55.96	87.92
	3	No	Weighted-softmax	56.37	56.41	50.14	50.09	62.27	90.49
	4	No	Center with weighted-softmax	56.90	57.06	50.74	50.71	62.24	90.33
	5	No	Weighted-cluster with weighted-softmax	56.27	56.42	50.03	49.97	61.57	90.10
	6	Yes	Softmax	52.22	49.51	45.4	44.54	63.27	90.75
	7	Yes	Center with softmax	47.08	40.02	39.51	38.27	54.91	86.75
	8	Yes	Weighted-softmax	59.72	59.72	53.97	53.93	66.47	91.85
	9	Yes	Center with weighted-softmax	59.60	59.50	53.83	53.82	65.35	91.21
	10	Yes	Weighted-cluster with weighted-softmax (proposed)	60.70	60.49	55.09	55.06	66.55	91.82

**Table 6:** Recognition performance of ResNet-50-based models on the test set.

Base Model	Model No.	Pre-Trained	Loss Function	Accuracy	F1-score	Kappa	Alpha	AUCPR	AUC
ResNet-50	11	No	Softmax	49.85	46.67	42.69	41.46	62.80	90.63
	12	No	Center with softmax	46.62	39.96	39.00	37.67	54.08	86.26
	13	No	Weighted-softmax	57.40	57.33	51.31	51.23	63.05	90.82
	14	No	Center with weighted-softmax	57.20	57.08	51.09	51.06	62.61	90.50
	15	No	Weighted-cluster with weighted-softmax	57.37	57.40	51.29	51.24	62.79	90.52
	16	Yes	Softmax	51.88	48.89	45.00	44.10	61.6	90.22
	17	Yes	Center with softmax	48.33	44.00	40.94	39.8	56.96	87.89
	18	Yes	Weighted-softmax	58.65	58.59	52.74	52.71	64.58	91.17
	19	Yes	Center with weighted-softmax	58.27	58.07	52.31	52.24	63.59	90.54
	20	Yes	Weighted-cluster with weighted-softmax	59.45	59.42	53.66	53.66	65.26	91.51
